# Curcumin: a new paradigm and therapeutic opportunity for the treatment of osteoarthritis: curcumin for osteoarthritis management

**DOI:** 10.1186/2193-1801-2-56

**Published:** 2013-02-18

**Authors:** Yves Henrotin, Fabian Priem, Ali Mobasheri

**Affiliations:** 1 Bone and Cartilage Research Unit, University of Liège, Institute of Pathology, Level +5, CHU Sart-Tilman, Liège, 4000 Belgium; 2 Physical Therapy and Rehabilitation Department, Vivalia, Princess Paola Hospital, Marche-en-Famenne, Belgium; 3 Bioxtract S.A., Parc Scientifique Créalys, Rue Guillaume Fouquet 30, Les Isnes, 5032 Belgium; 4 Musculoskeletal Research Group, Division of Veterinary Medicine, School of Veterinary Medicine and Science, Faculty of Medicine and Health Sciences, University of Nottingham, Sutton Bonington Campus, Sutton Bonington, Leicestershire, LE12 5RD UK

**Keywords:** Osteoarthritis, Curcumin, Bioavailability, Clinical trial

## Abstract

The management of osteoarthritis represents a real challenge. This complex and multi-factorial disease evolves over decades and requires not only the alleviation of symptoms, i.e. pain and joint function but also the preservation of articular structure without side effects. Nutraceuticals are good candidates for the management of OA due to their safety profile and potential efficacy. However, they are not part of the treatment guidelines and published recommendations. Curcumin is the yellow pigment isolated from the rhizomes of *Curcuma longa*, commonly known as turmeric. Curcumin is a highly pleiotropic molecule with an excellent safety profile. Strong molecular evidence has been published for its potency to target multiple inflammatory diseases. However, naturally occurring curcumin cannot achieve its optimum therapeutic outcomes due to its low solubility and poor bioavailability. Nevertheless, curcumin presents great potential for treating OA and has been categorized as having preclinical evidence of efficacy. This review aimed at gathering most of the available information to document the potential efficacy of curcumin based on the results obtained in in vitro models of cartilage and osteoarthritis and in other diseases.

## Introduction

The management of osteoarthritis (OA) is a challenge. As a multifactorial disease evolving over decades, OA is one of the most disabling rheumatic diseases. In addition, no cure has been discovered to date. There is growing interest in the medical management of OA. However, this area requires new therapeutic strategies and approaches to deal with OA in a rapidly growing elderly population.

The term “nutraceutical” is a combination of the words nutrition and pharmaceutical. It defines foods or food-derived products that provide health and medical benefits, including prevention and treatment (Ameye and Chee 2006; Henrotin et al. 2011; Kalra 2003).

The benefit of such products comes not only from their safety and efficacy but also by the fact that they are, by definition, and by legislation, devoid of adverse effects.

Curcumin is the yellow pigment isolated from the rhizomes of *Curcuma longa*, commonly known as turmeric (Henrotin et al. 2010; Shen and Ji 2012). Turmeric powder contains 5% curcumin, which is the main biologically active phytochemical compound. Curcumin exists as 2 tautomeric forms, keto and enol. The enol form is the most energetically stable in the solid phase or in solution. In solution, keto-enol tautomers are present. Curcumin extracts are composed of curcumin demethoxycurcumin, bisdemethoxycurcumin, 5^′^-methoxycurcumin and dihydrocurcumin (Kurien et al. 2007). The latest being both natural anti-oxidants (Masuda et al. 1999).

Curcumin is a highly pleiotropic molecule with an excellent safety profile. Strong molecular evidence has been published to support its potency for targeting multiple inflammatory diseases. However, naturally occurring curcumin cannot achieve its optimum therapeutic outcomes *in vivo* due to its low solubility and poor gastrointestinal absorption and systemic bioavailability.

## Method

Based on our previous review (Henrotin et al. 2010), curcumin represents great potential for the management of OA. In addition, our narrative review (Henrotin et al. 2011) categorized curcumin as having preclinical evidence of efficacy. The aim of the present review is to summarize most of the latest information regarding the therapeutic effects of curcumin in OA. A systematic search for articles with keywords “curcumin” and “arthritis” and “osteoarthritis” was performed in pubmed over the past five years. Recent clinical trials were search on http://www.clinicaltrials.gov. In addition, special attention was given to the pharmacokinetic issues associated with curcumin. Finally, data obtained with Arantal^®^ (BioXtract, Les Isnes, Belgium), high bioavailable turmeric extract in OA patients in a recent observational cohort study (OFKO survey) were discussed to illustrate the perspective of curcumin in treating OA patients.

### Clinical trials

A search on http://www.clinicaltrials.gov retrieved 75 clinical trials with “curcumin” and 35 with “turmeric”. Eleven trials used specifically turmeric extract. Taken together, it represented a total of 86 different clinical trials. Among them, 27 focused on curcumin in cancer therapy, 18 on curcumin in various inflammatory conditions including dermatitis, irritable bowel disease, colitis, 10 on curcumin in Alzheimer’s disease and different neurological conditions, 4 in rheumatology (including one in rheumatoid arthritis; two in OA; and one in fibromyalgia) and 6 in diabetes or metabolic syndrome. Ten of them aimed at studying various new formulations or curcumin pharmacokinetics. Finally the last eleven trials dealt with other conditions such as asthma, cardiovascular diseases or effect in healthy volunteers.

Two clinical trials dealt with OA and curcumin. The first one (ClinicalTrials.gov Identifier: NCT00792818) compared the effect of Curcuma Domestica Extracts (1500 mg/day divided into three times) to the one of ibuprofen (1200 mg/day divided into three times) in knee OA patients. This safety/efficacy study was based on the evaluation of pain on a WOMAC subscale after 28 days of treatment. This study was completed but no data have been available yet. The other trial dealing with OA and curcumin was the one with Arantal^®^, a highly bioavailable turmeric extract (ClinicalTrials.gov Identifier: NCT00992004). Its efficacy was evaluated on pain using a visual analog scale after 15 days of treatment. This study was completed but the data not yet divulgated. “Curcumin in Rheumatoid Arthritis” (ClinicalTrials.gov Identifier: NCT00752154) is a clinical trial registered on the Clinical Trials database. The study is sponsored by University of California at Los Angeles. It is a randomized, placebo-controlled crossover study in which 40 subjects will receive a total of 4 g of curcumin per day (capsule form, precise composition not disclosed) and then switch to placebo. The subjects’ participation may last up to 8 months. By completion of the study, all 40 subjects will have taken curcumin and placebo for 4 months each. Subjects will have blood tests, complete questionnaires, and be seen by the study doctor. At the present time status of this study is unknown and it looks like the original completion deadline will not be met and a larger and more comprehensive clinical trial may be necessary. However, when the study is completed it will be interesting to see if curcumin has provided any benefits for RA patients as this information will be useful for the design of future clinical trials of curcumin in OA.

These clinical trials illustrate the ongoing interest in curcumin and its applicability to a wide range of diseases.

### Systematic review

#### Therapeutic potential of curcumin

Curcumin has been used for centuries in traditional Chinese and Ayurvedic medicine for its anti-inflammatory properties (Goel et al. 2008). Turmeric has at least 53 different names in Sanskrit, each one referring to specific properties, including jawarantika, which destroys fever, mehagni, killer of fat, or rabhangavasa, which dissolve fat (Aggarwal 2010). Over the last few decades many scientific and clinical studies have focused on the potential of curcumin for treating various pathological conditions. Curcumin was investigated mainly for its anti-inflammatory and anti-oxidant potency. Oxidative damage and inflammation are now known to be a root cause of cancer and neurodegenerative diseases (Basnet and Skalko-Basnet 2011). Recently, these therapeutic effects have been reviewed in depth by Gupta et al. (Gupta et al. 2012b; Gupta et al. 2012a). Herein, a particular attention was paid to the potential therapeutic effects of curcumin on OA.

#### Curcumin in obesity, insulin resistance and diabetes

Obesity is a major risk factor for type 2 diabetes, atherosclerosis, cancer and other chronic diseases. Metabolic syndrome is defined as obesity, type 2 diabetes, hypertension and dyslipidemia (Aggarwal 2010). All these pathologies are closely linked to insulin resistance. This condition is closely related to OA. Indeed, OA has been shown to be induced, at least in part, by metabolic syndrome (Velasquez and Katz 2010). In addition, inflammation has been strictly linked to all these conditions (see (Aggarwal 2010) for review). Curcumin has been extensively studied as a treatment of obesity and obesity-related metabolic diseases (Aggarwal 2010). Curcumin was shown to directly interact with adipocytes, pancreatic cells, hepatic stellate cells, macrophages and muscle cells. Curcumin has been shown to reverse insulin-resistance, hyperlipidemia, hyperglycemia and other symptoms linked to obesity through the interaction with various key mediators, most of them are also involved in OA pathophysiology. Indeed, curcumin (5 μM) was shown to down-regulateTNF-.alpha; production in various tissues (Chan 1995). In addition, curcumin suppressed NF-κB through the inhibition of IκBα degradation at concentrations ranging from 2 to 60 μM (Singh and Aggarwal 1995) and reduced the NF-κB-regulated adipokines, including chemokines (MCP-1, MCP-4, eotaxin) at concentrations ranging from 0.1 to 10 μM (Woo et al. 2007) and interleukins (IL-1β, IL-6 and IL-8) at concentrations ranging from 5 to 20 μM (Wang et al. 2009). It also suppressed cyclooxygenase (COX)-2 and vascular endothelial growth factor (VEGF) through the inhibition of IKK activation (Aggarwal et al. 2006). Curcumin (20-40μM) was shown to be able to suppress plasminogen activator inhibitor type-I through the inhibition of the transcription factor early growth response (Egr)-1 gene product (Pendurthi and Rao 2000) and to down-regulate the secretion of insulin growth factor (IGF)-1 and to inhibit IGF-1 binding protein-3 (Xia et al. 2007). Furthermore, curcumin (10–50 μM) mimicked most of anti-diabetic drugs by activating PPARγ in hepatic stellate cells (Xu et al. 2003). It acted on cell signaling pathways by downregulating c-jun NH_2_ terminal kinase (JNK) at concentrations ranging from 5 to 20 μM (Wang et al. 2009). Curcumin (20 μM) also acts on developmental pathways by inhibiting the Wnt/β-catenin pathway in adipocytes (Jaiswal et al. 2002). This effect was demonstrated to occur with concentrations of 20 and 40 μM through the down-regulation of the transcription co-activator p300 (Ryu et al. 2008) or through the inhibition of the glycogen synthase kinase (GSK) 3β responsible of β-catenin phosphorylation at concentrations of 1 and 10 μM (Bustanji et al. 2009). Finally, curcumin (5–30 μM) was shown to interrupt leptin signaling by reducing the phosphorylation levels of leptin receptor (Ob-R) and downstream targets (Tang et al. 2009) and it increased the expression of adiponectin, which negatively regulates obesity when supplied as dietary curcumin in obese mice (Weisberg et al. 2008). Thus curcumin acts directly and strongly in mechanisms that sustain obesity and inflammation. In addition, most of these signaling intermediates and mediators also play key role in OA pathophysiology.

#### Potential therapeutic effects of curcumin in osteoarthritis

Many evidences support the use of curcumin in OA (Shen et al. 2012). Most of the biological effects observed and published for curcumin in chondrocytes and OA between 2002 and 2009 were reported in our previous narrative review (Henrotin et al. 2010). The following section will present the most recent data reported for curcumin and OA.

### In vitro and *in vivo* mechanisms of action

#### Anti-inflammatory activity

The anti-inflammatory property of curcumin has been known for centuries. It has been investigated and explained by studies showing how curcumin acts on inflammatory pathways. Curcumin (50 μM) was shown to inhibit NF-κB activation and translocation induced by IL-1β and the consequent expression of NF-κB induced pro-inflammatory genes, COX-2 and VEGF (Csaki et al. 2009). The effects of curcumin have been documented through its impact on cell signaling. Another study has described its effect on signaling and its inhibitory potency in chondrocytes in agarose constructs (Chowdhury et al. 2008). In this culture model, curcumin (0.01-100 ng/ml) was used for its potency to inhibit activator protein (AP)-1 and was then shown to reverse the IL-1β stimulated production of nitric oxide (NO) and prostaglandin E_2_ (PGE_2_).

The effect of curcumin (1–20 μM) has been also tested in vitro on human articular chondrocytes in alginate beads (Mathy-Hartert et al. 2009). This in vitro study demonstrated no toxic effect of curcumin on cell viability. Furthermore, it was able to produce an anti-inflammatory effect by inhibiting the pro-inflammatory mediators, i.e. PGE_2_, NO, IL-6 and IL-8. In bovine chondrocytes in monolayer, the biological activity of indomethacin, celecoxib and curcumin have been compared. Interestingly, curcumin but not non-steroidal anti-inflammatory drugs inhibited NO production. As anticipated, curcumin was less effective on PGE_2_ (IC50 = 2.5μM) production than NSAIDs (IC50 < 2.5 μM). However, in contrast to NSAIDs, curcumin inhibited COX-2, but not COX-1 gene expression (Mathy et al. 2007).

The anti-inflammatory potency of curcumin was also demonstrated in another connective tissue cell type. Curcumin (5μM) was shown to modulate inflammation in human tenocytes (Buhrmann et al. 2011) by the inhibition of COX-2 through its effect on NF-κB and on other related and equally important cell signaling pathway - the phosphatidylinositol-3 kinase/Akt pathway. The shared lineage and phenotypic similarities between cartilage and tendon cells suggest that curcumin would have similar effects on these connective tissues.

#### Anti-catabolic/anabolic effects

The anti-inflammatory potency of curcumin has been revealed by its ability to inhibit NF-κB activation, thus producing anti-catabolic effects. The inhibition of NF-κB by curcumin (50 μM) had significant effect on NF-κB-induced matrix degradation gene products (Csaki et al. 2009). This effect was shown in a study of a combination treatment with curcumin and resveratrol. Resveratrol or trans-3,5,4^′^-trihydroxystibene is a polyphenolic, antifungal natural phytoalexin found in grapevines (*Vitis vinifera*) and a variety of other plants and has been shown to possess potent anti-inflammatory and anti-oxidant properties. Curcumin alone or in combination with resveratrol reversed type II collagen inhibition induced by IL-1β. The combination treatment with resveratrol produced a synergistic effect. Another study revealed that curcumin (50 μM) produced anti-catabolic effect by the inhibition of MMP-9 through the inhibition of NF-κB activation (Shakibaei et al. 2007). The previously mentioned work with highly bioavailable curcumin (Mathy-Hartert et al. 2009) confirmed the anti-catabolic potency of curcumin in human chondrocytes by the inhibition of MMP-3, but failed to modify aggrecan production. In addition, curcumin (0.1-100 μM) tested on equine cartilage explants stimulated with IL-1β (10–25 ng/ml) showed the suppression of glycosaminoglycan (GAG) release (Clutterbuck et al. 2009). Anti-catabolic effects were also demonstrated in tenocytes (Buhrmann et al. 2011) by the inhibition of several matrix metalloproteinase (MMP) synthesis (MMP-1, MMP-9 and MMP-13) induced by IL-1β. Thus curcumin might be able to reverse the imbalance between anabolic and catabolic factors that occur in OA joint tissues by reducing the catabolic part. By this way, curcumin could prevent cartilage degradation and promote the accumulation in the extracellular matrix of newly synthesized matrix components like aggrecan.

The effects of curcumin have also been studied in adult mesenchymal stem cells. Curcumin (5 μM) was demonstrated to promote chondrogenesis of mesenchymal stem cells maintained in a high-density co-culture microenvironment by antagonizing the effects of pro-inflammatory cytokines (Buhrmann et al. 2010). All together these results support the potential structural and functional effects that curcumin could exert on joint tissues in OA.

#### Effect on cell survival and anti-apoptotic potency

Another important feature of curcumin in OA is its effect on cell survival. Indeed, it was not only proven to be safe for chondrocytes (Mathy-Hartert et al. 2009) but also shown to counteract the cytotoxic effect induced by IL-1β (Csaki et al. 2009). It was able to influence the changes that occur in mitochondrial such as swelling due to IL-1β stimulation and apoptosis. Curcumin reduced the apoptotic features induced by IL-1β. In addition, it stimulated anti-apoptotic factors (Bcl-2, Bcl-xL and TRAF1) and inhibited pro-apoptotic factors (caspase-3) (Csaki et al. 2009).

#### Clinical efficacy

Only a few clinical studies have been published with curcumin. One study tested the clinical efficacy of a herbomineral formulation containing a component rich in curcumin in subjects with OA (42 patients) in a one-month randomized, double-blind, placebo-controlled, cross-over study (Kulkarni et al. 1991). Treated group reported a positive effect on pain and mobility. More recently, the clinical efficacy of curcumin was tested in OA patients receiving Meriva^®^, a patented complex with phosphatidylcholine that improved curcumin bioavailability (Belcaro et al. 2010). Based on a previous study over 50 OA patients showing that Meriva^®^ improved joint pain and function, this study investigated efficacy and safety of the compound on a longer term (8 months). The evaluation included the measurement of several markers of inflammation (IL-1β, IL-6, sCD40L, sVCAM-1, ESR). One hundred OA patients were included in this study. They fulfilled the criteria for primary knee OA (grade 1 or 2). Patients were divided into 2 groups, one receiving “the best available treatment” as defined by patient’s general practitioner and specialists and the other receiving “the best available treatment and Meriva^®^”. In this last group, patients received two 500 mg Meriva tablets daily, one after breakfast and one after dinner (1000 mg/day corresponded to 200 mg curcuminoids/day). The composition of the Meriva^®^ tablets was a natural curcuminoid mixture (20%), phosphatidylcholine (40%), and microcrystalline cellulose (40%). The composition of the curcuminoids mixture was 75% curcumin, 15% demethoxycurcumin and 10% bisdemethoxycurcumin. Meriva^®^ significantly reduced pain and stiffness and improved joint function. All WOMAC scores were improved by the treatment with Meriva^®^, including social and emotional function. The improvement of physical function was also noteworthy. Finally, the markers of inflammation were significantly decreased in the treatment group between enrollment and after 8 months of treatment.

Curcumin was tested in patients suffering rheumatoid arthritis (Chandran and Goel 2012). In addition to be safe and not related to any adverse events, curcumin (500 mg) was the most effective to improve disease activity score (DAS) and American College of Rheumatology (ACR) score. Curcumin was administered alone or in combination with diclofenac sodium (50 mg).

Despite the paucity of published clinical data on curcumin and the overall poor quality of the trials, there is scope for promising future studies on curcumin in OA. However, since the in vitro effect was so well-documented and proven, the clinical efficacy needs to be further studied in OA patients. To this aim, one may keep in mind the importance of well-designed trials. Nevertheless, it also remain to undercover the precise mechanism of action of curcumin and to explain how it could achieve the effects that have been observed in vitro with supra-physiological concentrations, in human where its bioavailability could represent a challenge. Finally, this is important to note that curcumin when used in vitro, was dissolved in ethanol, acetone or dimethyl sulfoxide (DMSO).

### Pharmacokinetic issues

#### Absorption and distribution

Globally the pharmacokinetic profile of curcumin is characterized by a low serum concentration and a limited tissue distribution (Aggarwal and Sung 2009; Goel et al. 2008; Anand et al. 2007; Sharma et al. 2005; Dhillon et al. 2008). The available clinical data supports this notion. The serum concentration of curcumin peaks 1-2h after an oral dose in human subjects with peak serum concentrations of 0.5, 0.6 and 1.8 μM at massive doses of 4, 6 and 8 g/day (Cheng et al. 2001). Curcumin glucuronide and sulfate conjugates can be detected in plasma after oral administration (Vareed et al. 2008). Plasma curcumin, curcumin sulfate and curcumin glucuronide concentrations were in the range of 10 nM 1h after a large 3.6 g dose of oral curcumin (Sharma et al. 2004). Curcumin and its glucuronidated and sulfated metabolites can be measured in urine after a dose of 3.6 g/day (Heath et al. 2003) but cannot be detected in plasma at a dose lower than 3.6 g/day. Pure curcumin (2000 mg) administered to fasting healthy volunteers showed low curcumin concentration in plasma (<10 ng/ml) observed 1h after consumption (Shoba et al. 1998). High doses (8000 mg) of curcumin administered daily for 3 months to patients with pre-invasive malignant or high-risk pre-malignant conditions showed peak serum concentrations (1.75 μM) achieved after 1-2h oral intake and gradual decline of the levels within 12h (Cheng et al. 2001).

A pilot study with 15 patients with advanced colorectal cancer used standardized oral curcumin extract (each capsule contained 20 mg of curcuminoids (18 mg of curcumin and 2 mg of desmethoxycurcumin) suspended in 200 mg of essential oils derived from *Curcuma* spp.) daily up to 4 months. This study showed no toxicity but no evidence of detectable systemic bioavailability was provided either (Sharma et al. 2001). A curcuminoid formulation (each capsule contained 500 mg: 450 mg of curcumin, 40 mg of desmethoxycurcumin and 10 mg of bisdesmethoxycurcumin) administered orally for up to 4 months and allowing a rapid dose escalation that equated to curcumin doses between 450 and 3,600 mg daily showed that the later daily dose (3,600 mg) resulted in detectable levels of the compound and its conjugates in plasma. It was also present in urine (0.1-1.3 μM curcumin; 19–45 nM curcumin sulfate; 210–510 nM curcumin glucuronide). However, it was not detectable in patients who received doses less than 3,600 mg daily (Sharma et al. 2004). Finally high doses (12 g/day) of curcumin in phase I preclinical trial reinforces the low systemic bioavailability (Lao et al. 2006).

Interestingly, curcumin has been shown to accumulate in gastrointestinal tissues. Detectable levels of curcumin were found in both malignant (patients undergoing colorectal cancer surgery) and normal colorectal tissues after 7 days of curcumin 450, 1,800 or 3,600 mg/day orally (Garcea et al. 2005). The concentration of curcumin in normal tissue was 12.7 nmol/g of tissue, and in malignant colorectal tissue, 7.7 nmol/g of tissue. Curcumin sulfate and glucuronide were identified in these tissues. Low nanomolar levels of curcumin and its metabolites were measured in peripheral blood samples taken 1h after seventh dose of curcumin and in portal blood samples taken 6-7h after seventh dose of curcumin. However, parent drug was not detected in liver tissue of liver metastases of colorectal cancer but trace of the metabolic products (Garcea et al. 2004). A recent pilot study has demonstrated that after 14-days treatment with 2.35 g of curcumin-C3-complex, curcuminoids were detectable in all 23 participants biopsies (mean value 48.4 μg/g) but failed to induce deleterious effects on colonic mucosa (Irving et al. 2012).

New treatments with naturally occurring curcumin in its original form represent a major concern due to its low bioavailability. After oral ingestion, very little may actually reach the systemic circulation and even less may reach joint tissues. The dose of oral curcumin required to produce hepatic levels sufficient to produce pharmacologic activity is not feasible in humans with this pharmaceutical formulation (Basnet and Skalko-Basnet 2011). An important question addressed by a recent review (Shen and Ji, 2012) is “how can curcumin be potent in the face of low bioavailability”. There is a need for improvement of the formulation of curcumin and/or delivery system and many attempts have been made (Padhye et al. 2010). To bypass the intestinal metabolic enzymes, curcumin can be dissolved in oil before ingestion. Dissolution in oil produces no modification of its structure. It can then be directly absorbed into chylomicrons and subsequently into the lymphatic system then bypassing the liver and “the first pass phenomenon” (Anand et al. 2007). In this context it is important to note that the turmeric used in Indian culinary traditions is initially heated and dissolved in oil. Another attempt was made by the co-administration of curcumin and piperine (Shoba et al. 1998). Indeed, piperine, as an inhibitor of hepatic and intestinal glucuronidation enhanced the serum concentration and bioavailability of curcumin in both humans and animals.

Other solutions to improve bioavailability could come from liposomal delivery system (Basnet et al. 2012), nanoparticles (Anand et al. 2010) or other drug delivery systems (Bansal et al. 2011). The other possibility could tend to counteract curcumin metabolism by the intra-articular injection of high doses of curcumin to reach concentrations producing in vitro effects (Henrotin et al. 2010). Thus far there are no studies that have attempted intra-articular injection of high doses of curcumin. Suitable large animal models are required for such studies.

Finally, the improvement of the bioavailability of curcumin is a challenge. It could be improved by different techniques, such as heat-treatment (Kurien et al. 2007) or solubilized in dilute alkali (Kurien and Scofield 2007).

**Figure 1:**
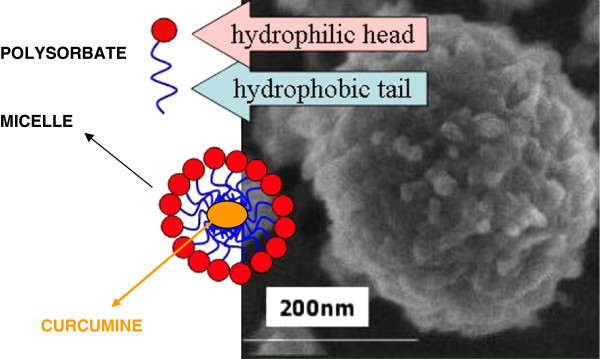
**Arantal**^**®**^**, a highly bioavailable curcumin.**

Highly bioavailable formulation of curcumin has been developped. Arantal^®^ is an innovative and patented formulation of curcumin, designed to increase the bioavailability of the active component curcumin in human (Figure 1). Bioavailability improvement was based on the increase of curcumin solubility in duodenal conditions; higher solubility was achieved by the pre-formulation of potential micro-emulsions, obtained in the digestive system (stomach, intestine) after disintegration of the capsule. The ideal conditions for the formation of micro-emulsions were made possible thanks to the presence of a high HLB (Hydrophilic-Lipophilic Balance) emulsifier: Polysorbate 80 (Tween 80^®^) combined to curcumin in a well-defined ratio. To improve the stability of the active part in the capsule, the presence of a weak acid was essential. The adjunction of little amount of turmeric essential oil during the micro-emulsion process still increased the solubility of curcumin. In this formulation, the solubility of curcumin in duodenal conditions (pH 6.8, 37°C) has been multiplied by a factor of 7.500, compared to the same amount of “native” curcumin.

A Phase I pharmacokinetics study on Arantal^®^, a high bioavailable turmeric extract with a water solubility increased 4000 times was run on 2 groups of 12 healthy individuals. The AUC obtained with Arantal^®^ is compared to the one of other formulations of curcumin (Table 1). Each group received orally 1 (42 mg curcumin) or 2 capsules (84 mg of curcumin) of Arantal^®^ respectively. With 2 capsules administered orally, the mean of Cmax on 12 individuals was 0.9 μM, with a statistical extrapolation at 1.6 μM with 4 capsules (administering 84 mg and 168 mg of curcumin respectively). These values were obtained after hydrolysis of conjugated metabolites by means β-glucuronidase and arylsulfatase (personal communication according to the analysis of the report performed by Advanced Technology Corporation M1060 Advanced provided by BioXtract S.A.; unpublished data).

**Table 1:** **Comparison of the bioavailability of different formulations of Curcumin based on their AUC (ng/ml)**

Curcumin	Meriva^®^ (376 mg)	Arantal^®^ 2 caps	***Arantal***^***®***^***4 caps (Extrapolated)***	Free curcumin 1800 mg Soria = 300 mg free curcumin
**AUC(ng/mL)**	538 ± 130.7	1938.34 ± 1090	*Between 3.500 and 4.000*	122.5 ± 29.3

AUC: area under the curve.

#### Metabolism

The metabolism of curcumin after oral administration is rapid and the consequent bioavailability in systemic circulation is low (Ireson et al. 2002). Curcumin is conjugated to produce curcumin glucuronides and sulfates or it is reduced to hexahydrocurcumin in the liver or in the intestine. The intra-peritoneal or systemic administration leads to the reduction of curcumin into tetrahydrocurcumin, hexahydrocurcumin and octahydrocurcumin (Ireson et al. 2002; Garcea et al. 2005). Curcumin has lower stability and can degrade under physiological conditions. The degradation products have been identified as trans-6-(4^′^-hydroxy-3^′^-methoxyphenyl)-2,4-dioxo-5-hexenal, ferulic acid, feruloyl methane and vanillin. The metabolic derivatives of curcumin do not possess the same biological activities as the original compound and were shown to be biologically inactive (Sandur et al. 2007; Ireson et al. 2001; Pan et al. 2000). However, some of its degradative products as ferulic acid or vanillin can be associated with anti-oxidant activity (Hatcher et al. 2008). In addition, the low stability of curcumin in aqueous solution is due to its hydrophobic nature (Aggarwal and Sung 2009). On the contrary, its degradative products have greater aqueous solubility than curcumin itself.

#### Elimination

Pharmacokinetic information was also obtained from animal models. Curcumin (1000 mg/kg) administered orally to rats showed excretion of 75% of the administered dose in the faeces and negligible amount detected in urine (Wahlstrom and Blennow 1978). The majority of curcumin ingested by rats is excreted in faeces (35% unchanged and 65% as metabolites) (Ravindranath and Chandrasekhara 1981). Curcumin is absorbed via the intestine with possible enterohepatic recirculation.

### Arantal^®^ : a high bioavailable turmeric extract

Arantal^®^ (Brand name, Flexofytol^®^, Tilman S.A., Somme-Leuze, Belgium), the high bioavailable turmeric extract described above, was evaluated in an observational cohort study, the OFKO survey (personal communication according to the report DIM 3 provided by BioXtract S.A. unpublished data). This study was fully sponsored and conducted by the manufacturer of Arantal^®^ (Tilman S.A.). 1463 patients were recruited. Among them, 1077 patients suffering from OA (64.6 ± 12.4; mean ± SD) were included in the study. 99.2% of the patients were taking NSAID and among them 36% were polymedicated. Otherwise, polymedication consisted in glucosamine sulfate (10.5%) with or without chondroitin sulfate (alone 0.8% or in combination 22.2%), type II collagen (2.5%), intra-articular hyaluronic acid (4.3%) or corticosteroids (1.6%). Interestingly only 1% of patients were using acetaminophen. Patients were asked to take 4 capsules of Flexofytol^®^ (2x2 capsules/day or 2 x 84 mg of curcumin) maintaining their medication for 3 months. Pain was evaluated using VAS on 3 visits throughout the study. Flexofytol^®^ was well tolerated. The reported side effects were very rare and minor (19 patients reported adverse effects - essentially nausea and bloating). 93.45% of the patients decided to remain on Flexofytol^®^ after the first visit, among them 70.56% maintain also the 4 capsules a day. Results revealed a significant pain reduction of 48% over the 3 visits in the group of 1077 OA patients, with a mean reduction of 32.45mm. This effect improved over time. Of special note, MCII (minimum clinical important improvement corresponding to a decrease of 19mm on the VAS scale) was obtained for 81% of patients. In addition, a majority of patients using Flexofytol^®^ reported that they didn’t need any more any other concomitant medications (only 9% of patients kept NSAIDs, 16% other drugs and 85% acetaminophen). 93.45% of patients decided to maintain Flexofytol^®^ in their medication for OA after the study ended due to their overall satisfaction with the product.

## Conclusion

Nutraceuticals are not part of the published OARSI and EULAR recommendations for the management of OA (Zhang et al. 2008,2007; Zhang et al. 2010; Jordan et al. 2003; Zhang et al. 2005). The promising in vitro results and the interesting clinical observations gathered here for curcumin should refocus efforts to develop therapies based on new formulations of curcumin. Well-designed clinical studies are needed to determine and document the efficacy of curcumin and combination products with curcumin in OA patients. Due to the increased bioavailability obtained with certain formulations, we expect improved bioavailability and consequently enhanced clinical efficacy. New formulations of curcumin should demonstrate improved pharmacokinetic and clinical efficacy. Curcumin is still worthy of our interest and research efforts and needs to be further studied in clinical trials.

Hence, curcumin represents a new paradigm since it is not yet a recommended intervention in OA but should be considered based on its safety and efficacy. In addition, taken altogether, these data highlight the needs in OA research for the near future as good quality and well-designed trials.

## Abbreviations

ACR: American college of rheumatology
AP: Activator protein
COX: Cyclooxygenase
DAS: Disease activity score
Egr: Early growth response
GAG: Glycosaminoglycan
GSK: Glycogen synthase kinase
IGF: Insulin growth factor
JNK: c-jun-NH_2_ terminal kinase
IL: Interleukin
MMP: Matrix metalloprotease
NO: Nitric oxide
OA: Osteoarthritis
Ob-R: Leptin receptor
PG: Prostaglandin
VEGF: Vascular endothelial growth factor
